# Femoral vein obturator bypass revascularization in groin infectious bleeding: two case reports and review of the literature

**DOI:** 10.1186/1752-1947-7-75

**Published:** 2013-03-18

**Authors:** Albert Busch, Udo Lorenz, George Christian Tiurbe, Christoph Bühler, Richard Kellersmann

**Affiliations:** 1 Department of General, Visceral, Vascular and Paediatric Surgery University Clinic of Würzburg, Oberdürrbacher Strasse 6, Würzburg, D-97080, Germany

**Keywords:** Obturator bypass, Groin infection, Autologous, Extra-anatomical, Femoral vein

## Abstract

**Introduction:**

Groin infections resulting in arterial bleeding due to bacterial vessel destruction are a severe challenge in vascular surgery. Patients with them most often present as emergencies and therefore need individualized reconstruction solutions.

**Case presentation:**

Case 1 is a 67-year-old man with infectious bleeding after an autologous reconstruction of the femoral bifurcation with greater saphenous vein due to infection of a bovine pericard patch after thrombendarterectomy. Case 2 is a 35-year-old male drug addict and had severe femoral bleeding and infection after repeated intravenous and intra-arterial substance abuse. Both patients were treated with an autologous obturator bypass of the superficial femoral vein. We review the current literature and highlight our therapeutic concept of this clinical entity.

**Conclusions:**

Treatment should include systemic antibiotic medication, surgical control of the infectious site, revascularization and soft tissue repair. An extra-anatomical obturator bypass with autologous superficial femoral vein should be considered as the safest revascularization procedure in infections caused by highly pathogenic bacteria.

## Introduction

Severe groin infections with inguinal blood vessel destruction may be caused by intravenous substance abuse, radiation scars, transfemoral interventions and, most commonly, infection of prosthetic vascular implants (incidence 2% to 18%) [1-4]. Complications include life-threatening bleeding, acute ischemia, septic embolization and systemic sepsis. Finding the appropriate strategy for each patient remains an individual challenge.

## Case presentation

### Case 1

**Figure 1:**
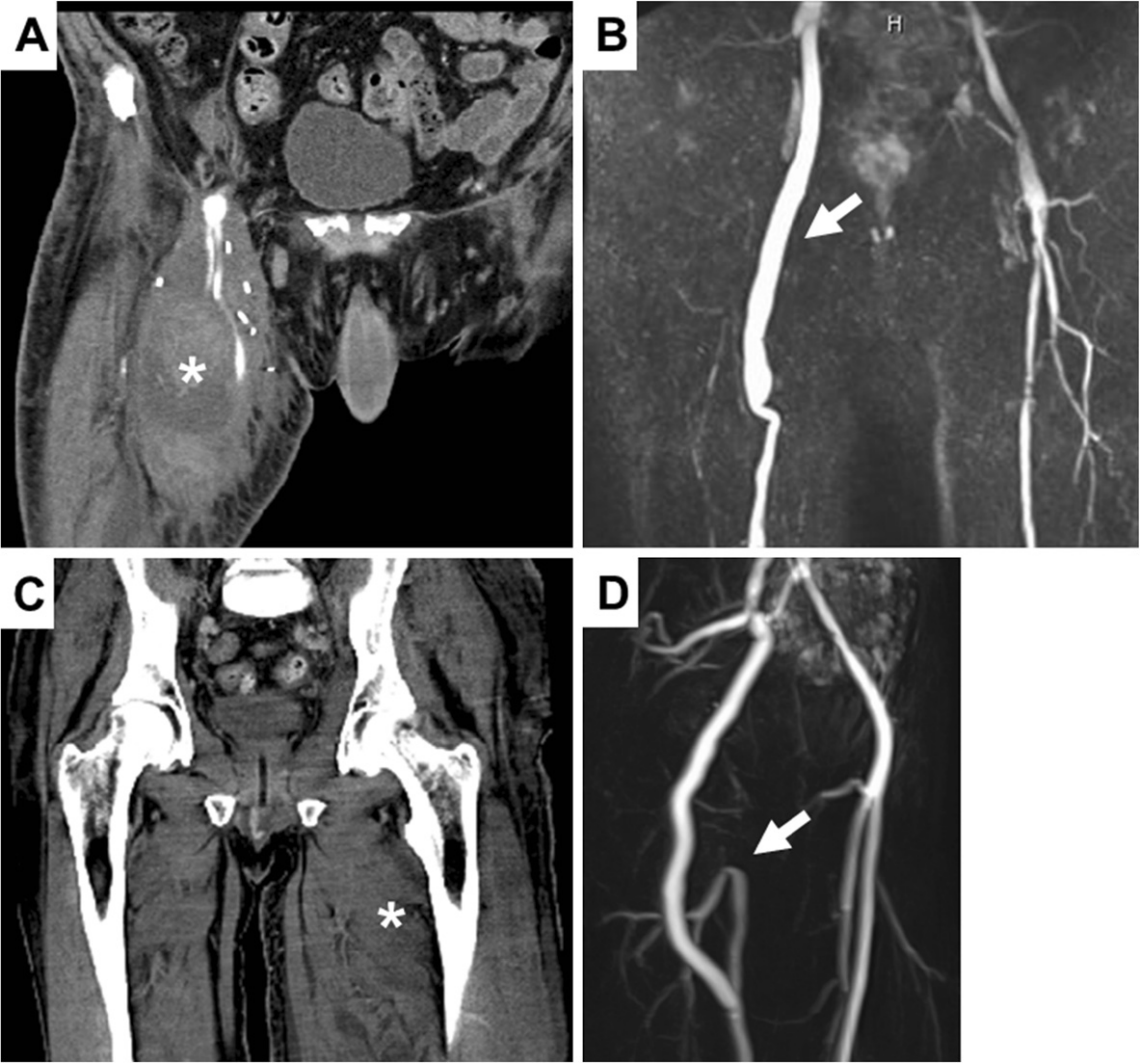
**(A) Computed tomography scan of case 1 obtained after rebleeding due to septic pseudoaneurysm showing liquid, putrid formation (asterisk) with arterial extravasation caused by erosion of the distal anastomosis of the greater saphenous vein interposition and extensive perifocal soft-tissue edema in the right upper thigh.** (**B**) Magnetic resonance angiogram of the obturator bypass in case 1 nearly 3 years after bypass surgery showing bypass perfusion free of stenosis (arrow). Case 2 (**C**) Computed tomography scan of case 2 at time of admission showing swelling, subcutaneous purulent formation and perifocal edema (asterisk). (**D**) Magnetic resonance angiogram obtained 3 months after surgery in case 2 showing the obturator bypass with retrograde perfusion of the profundal femoral artery by additional end-to-end anastomosis with proximal superficial femoral artery (arrow).

A 67-year-old Caucasian man was admitted to our institution with infectious bleeding in the right groin and lower limb ischemia 4 months after a prolonged hospitalization for common femoral artery thrombendarterectomy with a bovine pericard patch plasty and successful antibiotic treatment due to postoperative methicillin-resistant *Staphylococcus aureus* (MRSA) superinfection of an inguinal lymphatic fistula. For surgical control of bleeding, orthotopic revascularization with iliacofemoral and iliacoprofundal greater saphenous vein interposition and resection of the xenogenic patch material was performed, accompanied by systemic MRSA-specific antibiotic treatment. Three weeks later rebleeding occurred due to an infectious arterial pseudoaneurysm. Intra-operative findings revealed complete erosion of the profundal venous graft anastomosis (Figure 1A).

### Case 2

**Figure 2:**
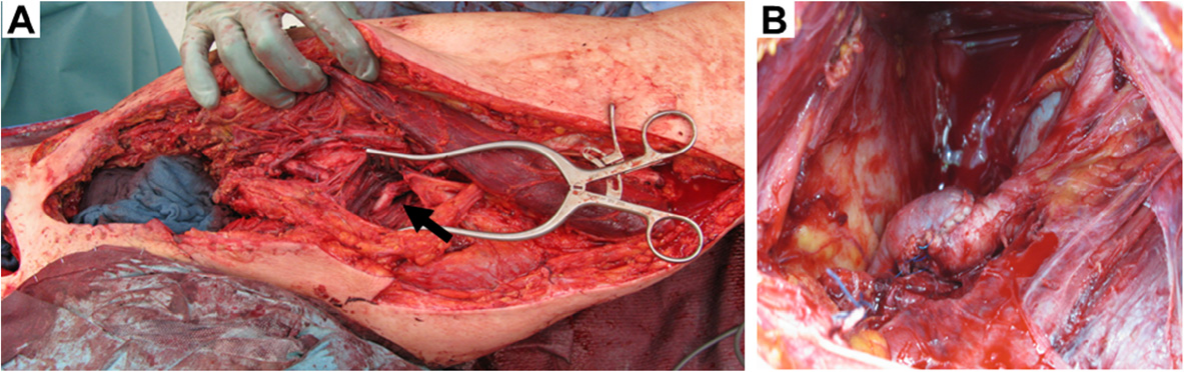
**Intra-operative images.** (**A**) The intraoperative site after radical debridement and the distal anastomosis of the implanted bypass (arrow) with gross incisions opening the whole upper thigh. (**B**) Suprapubic incision showing the proximal anastomosis before leaving the pelvic region through the obturator foramen.

A 35-year-old Caucasian man with extensive groin infection due to repeated drug abuse via the femoral vessels was transferred to our hospital 1 day after emergency implantation of an iliacofemoral and iliacoprofundal polytetrafluoroethylene prosthesis due to infectious bleeding together with rhabdomyolysis and sepsis with liver and kidney failure (Figure 1C). Massive gangrenous destruction of the femoral vessels and surrounding tissue were found intra-operatively, pending sufficient coverage of an orthotopic revascularization (Figure 2A). Group B *Streptococcus* was identified.

An obturator bypass using the ipsilateral superficial femoral vein to bypass the infected area extra-anatomically from the common iliac artery to the distal femoral artery was performed in both cases (Figures 2A and 2B). Venous graft was harvested from the non-infectious part of the thigh to the venous confluence. This procedure was accompanied by systemic antibiotic therapy, then by local debridements and finally by mesh graft augmentation. Physical therapy and 30mg enoxaparin daily were administered from day 1 after the operation. The patients were dismissed in good health and able to walk with regression of initial lymphatic swelling. Both bypasses remained patent during follow-up at 32 and 12 months in cases 1 and 2, respectively (Figures 1B and 1D). Venous function was not hindered clinically, with normal thigh circumference.

## Discussion

Though challenging, individual primary revascularization procedures, especially in young patients, should be considered the first treatment option because of high amputation rates after double or triple ligation (up to 27%) or severe claudication [4-7]. Without complete graft removal, reinfection occurs in about one-third of cases [8]. Insufficient soft-tissue coverage at the infection site weighs in favor of an extra-anatomic bypass configuration.

The obturator bypass, with primary patency rates of up to 76% after 2 years, has proven to be feasible in a few small clinical series with a considerable risk of late prosthetic graft infection remaining [1,3,4,9-11]. Therefore, evidence is increasing for the superiority of autologous revascularization with femoral veins to allograft, xenograft or synthetic materials, as well as greater saphenous vein (complete erosion despite antibiotic therapy in case 1) in terms of infection resistance, patency rates and availability [12-15].

To the best of our best knowledge, investigators of only one clinical series have reported the use of obturator bypass fashioned from autologous superficial femoral vein at infection sites [16]. We therefore want to highlight in our case presentations its potential benefits in vascular groin infections, with a crucial focus on the underlying bacteriology, such as MRSA and Group B *Streptococcus* in this report. With proven susceptibility of several native and prosthetic materials to MRSA and rising clinical incidence, femoral vein obturator bypass is the safest revascularization procedure with the best long-term results [17].

## Conclusions

The extra-anatomic obturator bypass with femoral vein is a safe and feasible revascularization procedure in patients with severe groin infections and highly pathogenic bacteria such as MRSA, *Pseudomonas aeruginosa* and Group B *Streptococcus*. It should be considered the primary treatment option by vascular surgeons confronted with this problem, especially in young patients.

## Consent

Written informed consent was obtained from the patients for publication of this case report and any accompanying images. A copy of the written consent is available for review by the Editor-in-Chief of this journal.

## Competing interests

The authors declare that they have no competing interests and did not receive any payment from industrial partners.

## Authors’ contributions

RK, CB, CT and AB performed surgery on the patients and were responsible for postoperative intensive medicine care. UL performed vascular graft infection studies in mice and was a major contributor in writing the manuscript. All authors read and approved the final manuscript.
